# Optimal Sites for Upper Extremity Amputation: Comparison Between Surgeons and Prosthetists

**DOI:** 10.3390/bioengineering12070765

**Published:** 2025-07-15

**Authors:** Brandon Apagüeño, Sara E. Munkwitz, Nicholas V. Mata, Christopher Alessia, Vasudev Vivekanand Nayak, Paulo G. Coelho, Natalia Fullerton

**Affiliations:** 1 University of Miami Miller School of Medicine, Miami, FL 33136, USA; 2 Hanger Clinic, Pembroke Pines, FL 33024, USA; 3 Department of Biochemistry and Molecular Biology, University of Miami Miller School of Medicine, Miami, FL 33136, USA; 4 Dr. John T. Macdonald Foundation Biomedical Nanotechnology Institute (BioNIUM), University of Miami, Miami, FL 33146, USA; 5 DeWitt Daughtry Family Department of Surgery, Division of Plastic Surgery, University of Miami Miller School of Medicine, Miami, FL 33136, USA

**Keywords:** amputation level, prosthetic, upper extremity, function

## Abstract

Upper extremity amputations significantly impact an individual’s physical capabilities, psychosocial well-being, and overall quality of life. The level at which an amputation is performed influences residual limb function, prosthetic compatibility, and long-term patient satisfaction. While surgical guidelines traditionally emphasize maximal limb preservation, prosthetists often advocate for amputation sites that optimize prosthetic fit and function, highlighting the need for a collaborative approach. This review examines the discrepancies between surgical and prosthetic recommendations for optimal amputation levels, from digit amputations to shoulder disarticulations, and explores their implications for prosthetic design, functionality, and patient outcomes. Various prosthetic options, including passive functional, body-powered, myoelectric, and hybrid devices, offer distinct advantages and limitations based on the level of amputation. Prosthetists emphasize the importance of residual limb length, not only for mechanical efficiency but also for achieving symmetry with the contralateral limb, minimizing discomfort, and enhancing control. Additionally, emerging technologies such as targeted muscle reinnervation (TMR) and advanced myoelectric prostheses are reshaping rehabilitation strategies, further underscoring the need for precise amputation planning. By integrating insights from both surgical and prosthetic perspectives, this review highlights the necessity of a multidisciplinary approach involving surgeons, prosthetists, rehabilitation specialists, and patients in the decision-making process. A greater emphasis on preoperative planning and interprofessional collaboration can improve prosthetic outcomes, reduce device rejection rates, and ultimately enhance the functional independence and well-being of individuals with upper extremity amputations.

## 1. Introduction

In 2005, an estimated 500,000 people in the U.S. were living with limb loss, a number expected to double by 2050 [1]. Upper extremity amputations are classified as major (above the wrist) or minor (below the wrist) based on the level of limb loss. Major amputations include interscapulothoracic, transhumeral, elbow disarticulation, transradial, and wrist disarticulation [2]. Minor amputations involve phalangeal, metacarpal, and carpal levels [3].

Upper extremity loss significantly impacts physical and psychosocial well-being, daily function, and quality of life [4,5,6]. Unlike lower limb amputations, upper limb loss presents with greater functional challenges, leading to higher disability ratings and lower satisfaction with prosthetic function [4,5,7,8,9]. Although prostheses improve quality of life, rejection rates remain high (30–80%) due to discomfort, limited durability, and inadequate functionality [10,11,12,13,14]. One known contributor to the utility and functional capabilities of an upper extremity prosthesis is the level at which the amputation is performed [15]. Precise and methodical determination of residual amputation stump length has been found to not only improve device proficiency but also to prevent subsequent surgical revisions that may be necessary to achieve an optimal length-to-function ratio [16].

Amputation level plays a crucial role in prosthetic utility and functionality. While general limb length guidelines exist, they often overlook manufacturer-specific variations and evolving prosthetic technology [16]. Surgeons are advised to collaborate with prosthetists to optimize amputation site selection, but this is not always standard practice. This review seeks to bridge the gap between surgical teachings and prosthetists’ recommendations by comparing optimal amputation levels and their compatibility with specific prosthetic devices.

## 2. Methods

For this review, a literature search using PubMed and Google Scholar was conducted. Studies published from database inception to March 2023 were assessed. Key words included ‘amputation’, ‘disarticulation’, ‘prosthesis’, ‘digit’, ‘hand’, ‘transradial’, ‘transhumeral’, and ‘shoulder’. Eligible sources included peer-reviewed studies, clinical guidelines, and systematic reviews, excluding animal studies and non-English publications. Two reviewers independently screened titles and abstracts, followed by a full-text review. Data extraction focused on surgical and prosthetist recommendations, functional outcomes, and prosthetic compatibility. All photographs provided herein were obtained from the archives of the senior author (N.F.). Informed consent was obtained from all subjects within the study.

## 3. Prosthetic Device Types

**Figure 1:**
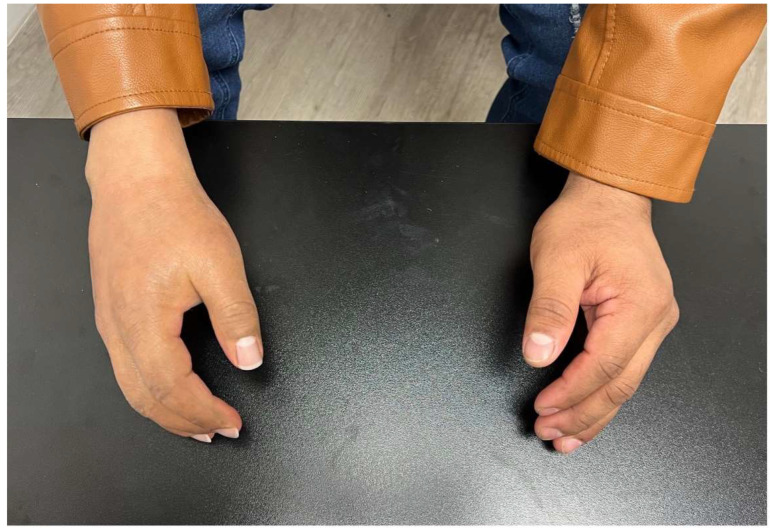
Silicone restoration of the right hand.

Multiple prosthetic devices exist, each with its own advantages and disadvantages (Table 1). Passive functional prosthetics (PFPs) enhance aesthetics and assist in simple tasks but provide minimal grasping function. However, they allow digit opposition for fixed grasp and partial digit extension for limited functionality [17]. They are lightweight and cosmetically resemble a natural hand (Figure 1).

Body-powered (BP) prostheses use cables and harnesses to control movement, offering durability, easy maintenance, and greater sensory feedback [18]. They provide a wider range of motion and superior strength for heavy lifting compared to myoelectric (MYO) devices, leading to improved functionality and patient satisfaction [19,20,21]. BP prostheses cost between USD 4000 and USD 10,000, making them more affordable than MYO devices, which can exceed USD 75,000 [22].

Myoelectric (MYO) prostheses use electromyographic (EMG) signals from muscle contractions in the residual limb to control movement of the terminal device [23]. They offer improved fine motor control and improved cosmesis, as they more closely resemble the natural anatomy [19,24,25]. These devices excel in precise movements and are better suited for light tasks that require movement accuracy rather than heavy lifting [20,26,27]. Although the evidence is mixed, MYO devices may also have a positive effect on phantom limb pain [28,29].

Hybrid prostheses combine the fine motor control of MYO devices with the durability of BP models, making them ideal for individuals needing adaptability across various activities [30,31]. They are particularly beneficial for above-elbow amputations, offering enhanced functionality in complex movements like lifting against resistance [32]. Novel hybrid approaches have been introduced, including a recently patented design which features a flexible, bionic hand with palm and finger assemblies with independent bending and transmission components [33]. By enabling coordinated finger movement via fewer actuators, the lightweight prosthesis improved dexterity and digit control [33]. Alternatively, three-dimensional (3D) printing has been employed to create a prosthetic finger with a monolithic structure and flexure hinge [34]. By treating the hinge as a 3D object, the design allowed for a soft and lightweight prosthesis that better mimicked the natural finger and improved user comfort [34].

## 4. Review of Surgeons’ Versus Prosthetists’ Recommended Amputation Lengths

### 4.1. Digit Amputation

#### 4.1.1. Surgical Recommendations

Finger and thumb amputations are the most common trauma-related amputations in emergency settings [35,36]. Thumb function is directly proportional to stump length, making length preservation critical [37,38]. Losing the thumb at the metacarpophalangeal joint results in a 40% loss of hand function and 36% of upper limb function [39]. Surgical goals focus on removing painful or nonviable tissue while preserving as much functional length as possible [37]. Neuroma formation should be prevented to avoid post-amputation pain [40].

For distal phalanx amputations, preserving the flexor digitorum superficialis (FDS) insertion helps maintain joint flexion [38]. A finger flap is recommended if the wound is ≥2 mm distal to the volar distal interphalangeal (DIP) joint skin crease, while shortening amputation is performed if both digital nerves are damaged [41]. Middle phalanx amputations proximal to the FDS insertion require proximal interphalangeal (PIP) joint disarticulation to prevent loss of motion control. Preserving articular cartilage reduces pain and enhances shock absorption [42]. Extensive central digit amputations may require ray amputation to narrow the residual gap and improve grip function [41].

#### 4.1.2. Prosthetist Recommendations

Similarly, prosthetists prioritize salvaging digit length to maintain pincer grasp. For thumb amputations, passive prosthetics (e.g., Vincent Passive Thumb, Titan Thumb, M-Thumb) enhance aesthetics, while BP prostheses (e.g., Thumb Driver by Naked Prosthetics) improve function in proximal amputations [43].

**Figure 2:**
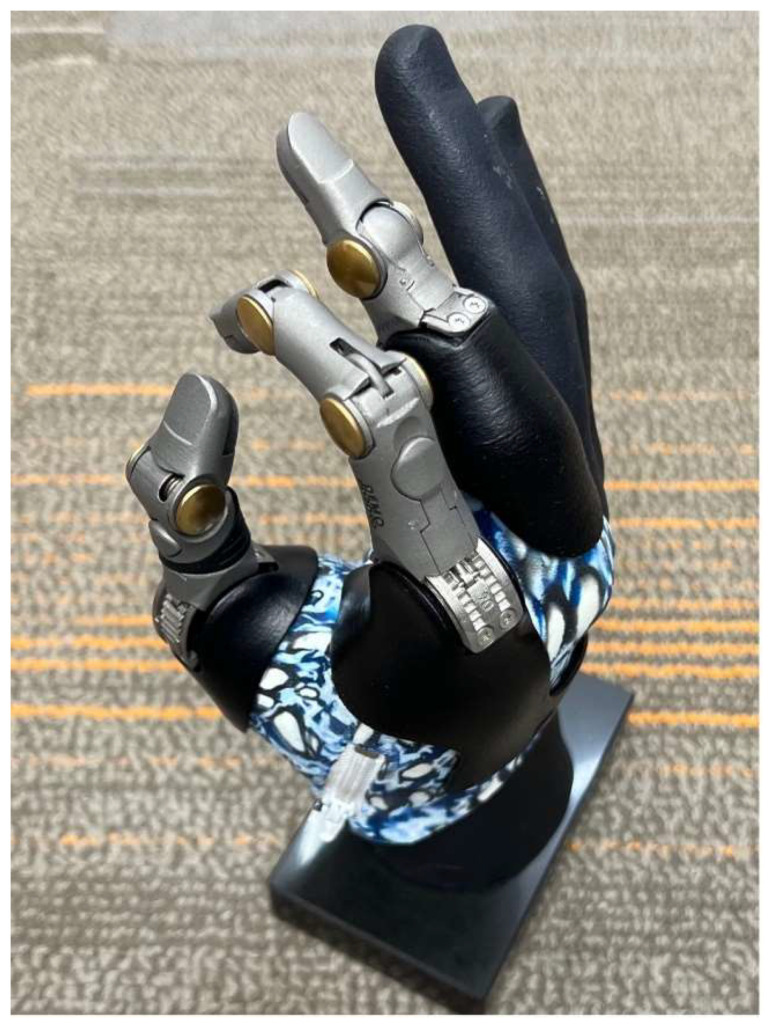
Passive functional prostheses of digits 1–3 at varying interphalangeal joints, allowing for passive ratcheting mechanism.

For amputations distal to the DIP joint, passive prosthetics (e.g., Regal Prosthesis Ltd., Livingskin) offer cosmetic benefits, improving social acceptance despite limited opposition [43]. At levels proximal to the DIP, passive devices (e.g., Point Partial, Point Digit, Grip Lock) remain options, though less cosmetic and without active prehension (Figure 2). At this level, BP prostheses (e.g., PIP Driver, MCP Driver, Partial M-Finger) become available and provide grasp function but are less aesthetically favorable. For either prosthetic type, preserving the joint is crucial to avoid length discrepancies with the contralateral hand [43]. To use a BP prosthesis, the amputation must leave a small segment distal to the joint to allow for engagement of a ring that flexes the prosthesis during movement.

### 4.2. Partial Hand/Transcarpal Amputation

#### 4.2.1. Surgical Recommendations

Preservation of the length of the metacarpals is recommended, as it allows the residual limb to function as a helper hand [41]. If dorsal wrist extensor insertions are removed, extensor tendons should be reinserted into the residual carpus [41]. In these cases, myocutaneous flaps are preferable to skin flaps for pressure relief, and the associated atrophy will facilitate the placement of passive finger prostheses to assist in grasp [17]. In severe cases involving the thumb or multiple digits, toe-to-hand transfer may be considered [41].

#### 4.2.2. Prosthetist Recommendations

If at least two opposable, sensate digits remain, preserving length is crucial to minimize graft and scar tissue. Thumb preservation is especially important to maintain sensation for grasp with the prosthesis. Techniques to save the thumb include bone lengthening, web deepening, or bone grafting.

Prosthetic options vary by amputation level. Distal amputations with minimal functional loss may benefit from passive functional prosthetics (e.g., Point Digit, Grip Lock, Titan Full, Vincent Passive) with a ratcheting flexion mechanism that locks the prosthetic into various degrees of flexion [40]. BP prosthetics (e.g., Robin-Aids, M-Fingers) are suitable for patients who require more durability and grasping ability for daily activities.

**Figure 3:**
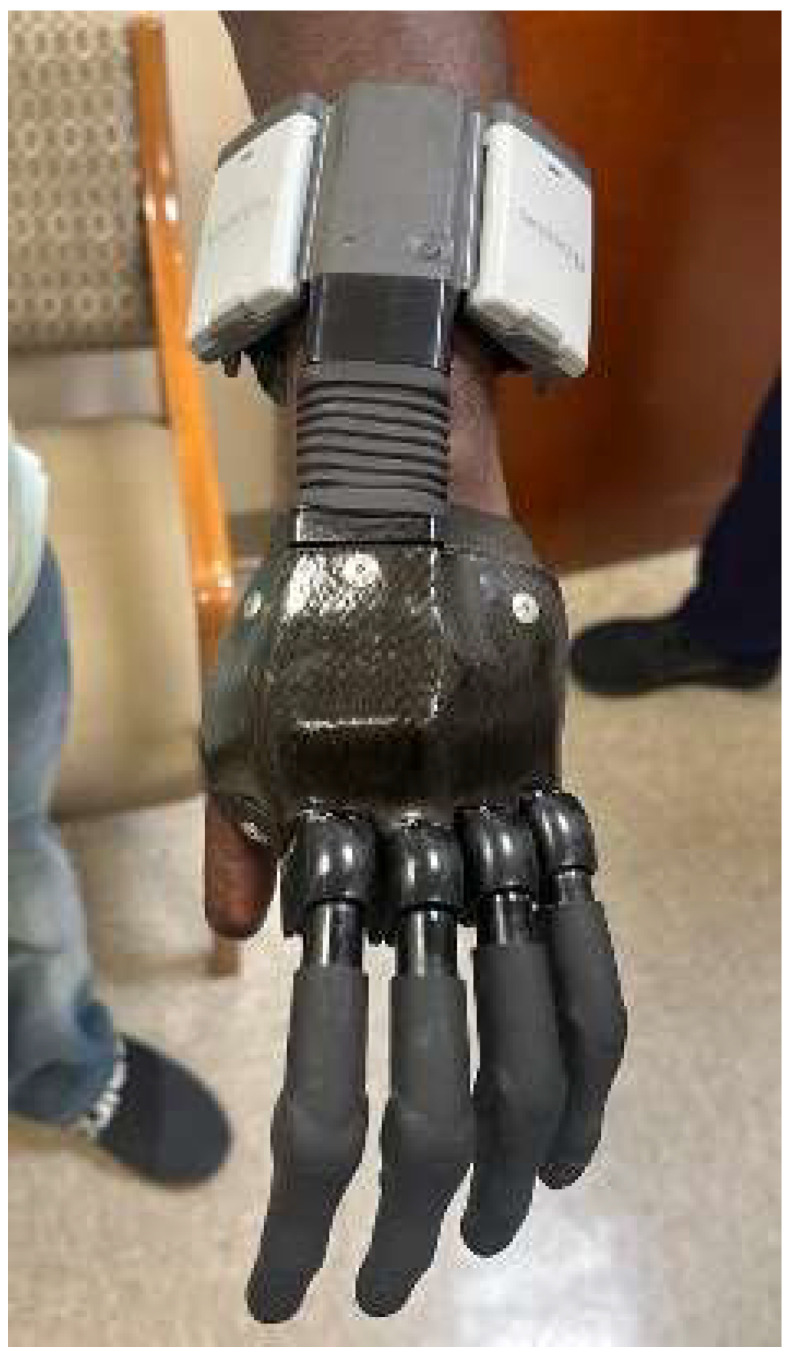
Transcarpal myoelectric prosthesis in patient who had undergone Starfish procedure.

For fine motor control and grasping, MYO prosthetics (e.g., Ossur iDigits, Vincent Partial Hand) are ideal (Figure 3) [43]. Barriers to effective MYO prosthetic control include nonintuitive control mechanisms, signal interference from the small hand muscles, and a limited number of muscle targets sufficiently close to the surface for the prosthetic [44]. The Starfish procedure addresses these issues by repositioning residual interosseous muscles to a more superficial area for better prosthetic control without adding bulk [44,45].

Recently, a machine learning-based approach has been used to improve prosthetic hand control [46]. By using reinforcement learning and computer vision, autonomous object grasping was improved without requiring extensive user training [46]. This highlights the potential for highly responsive prosthetics that reduce reliance on muscle signals.

### 4.3. Wrist Disarticulation

#### 4.3.1. Surgical Recommendations

Wrist disarticulations are preferred over proximal amputations when sufficient tissue allows for deep tissue and bony coverage [37]. Preserving the ulnar styloid stabilizes the distal radial ulnar joint and enables radial rotation [37]. Additionally, trimming the radial and ulnar styloid prevents pressure points and improves prosthetic fit while preserving the triangular fibrocartilage complex for pronation and supination. In preparation for prosthetic use, special consideration is given to the extensor tendon, as it is essential to maintain muscle tension and stabilization for MYO control of the terminal device [37]. Regarding the nerves, traditional options include burying the nerve in muscle or bone [41].

#### 4.3.2. Prosthetist Recommendations

Wrist disarticulation can be challenging due to limited low-profile wrist and hand components that maintain function and symmetrical limb lengths. Thus, transradial amputations are generally preferred [47]. If wrist disarticulation is necessary, prosthetists recommend a low-profile wrist unit or direct mount of the hand to the prosthesis at the wrist disarticulation level [47]. While this level may restrict supination and pronation, it allows for lower socket trim lines and suspension just proximal to the styloid.

### 4.4. Transradial Amputation

#### 4.4.1. Surgical Recommendations

In transradial amputations, forearm length is proportional to prosupination [41]. Amputating too close to the elbow limits this motion, so adequate length should be preserved when possible [41]. A minimum of 5 cm from the elbow is needed for prosthetic fitting, while 16–18 cm distal to the olecranon provides a longer lever arm, reducing residual limb stress [41,48]. Intraoperatively, the bicep tendon is transferred from the radius to the ulna to prevent flexion contracture [49]. Dividing the tendon at its radial tuberosity insertion can increase functional length, leaving the brachialis as the primary elbow flexor [41]. Rotary movement of the radius is preserved to prevent synostosis [49]. Myodesis, myoplasty, or myofascial closure stabilizes muscles to minimize stump pain [50]. Myoplasty of flexor and extensor muscles over the radius and ulna aids in muscle stabilization and facilitates later prosthetic fitting [50].

#### 4.4.2. Prosthetist Recommendations

**Figure 4:**
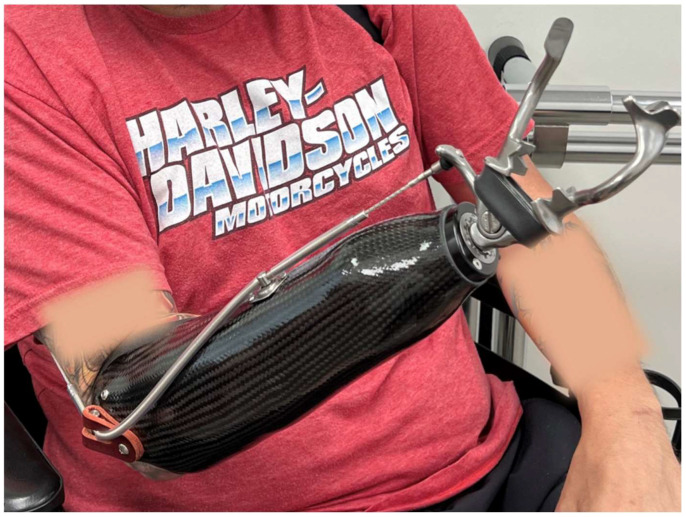
Transradial body-powered prosthetic.

**Figure 5:**
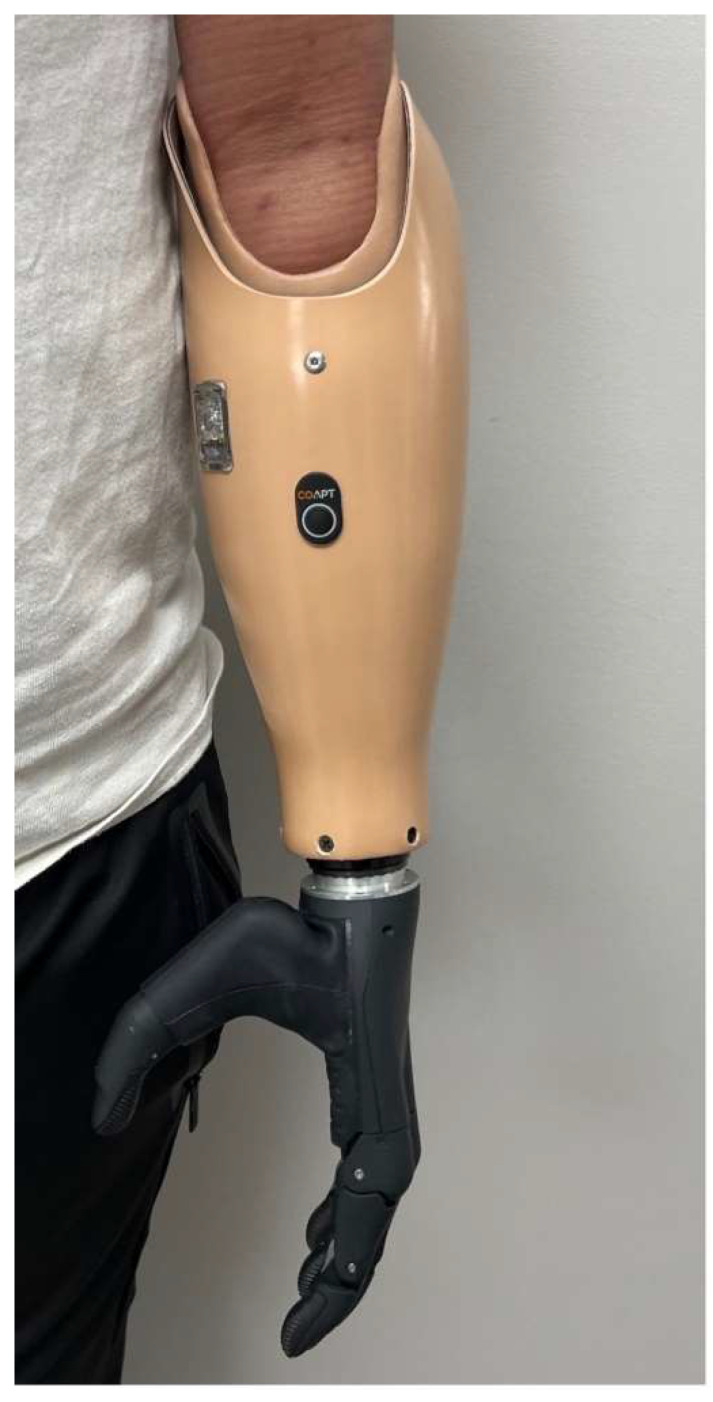
Transradial myoelectric prosthesis.

This level gives optimum outcomes for prosthetic fit as it allows for the fitting of all available BP (Figure 4) and MYO prosthetics (Figure 5). The socket must house batteries, controllers, electrodes, and wires for MYO function. Suspension is either suction or anatomically based, depending on patient needs. A stable limb volume is required for MYO prosthesis fitting. To determine the optimal length for amputation, surgeons should use the following formula [47]:

*Y* = *X* − 22.5 cm (1)

where Y is the optimal residual limb length (including soft tissue), X is the distance from the lateral epicondyle to the thumb tip (uninvolved limb, elbow at 90°, and 22.5 cm is the average prosthetic terminal device and wrist unit length.

Using this formula, the ulna should be 0.8–1.25 cm shorter than Y, and the radius 1.25–2.5 cm shorter than Y. Surgeons should also consider that shortening the residual limb too much can impair natural supination and pronation, potentially necessitating a prosthetic wrist unit to restore rotational function.

Although often overlooked, prosthetic length estimation must consider socket materials for function, skin protection, and user comfort [51]. Prosthetic socket materials (thermoplastic or high-consistency rubber silicone) add 1–2 cm, while wrist components add 1–2 cm, totaling 2–4 cm [52]. For this reason, it is preferred to have at least 4 cm of the ulna from the olecranon preserved. If the residual limb is shorter than this, a more proximal amputation is advised [48]. Table 2 provides total prosthesis length calculations. Considering these measurements ensures optimal amputation site selection for symmetry and function.

TMR, increasingly used in upper limb amputations, transfers residual mixed or sensory nerves to newly denervated muscles [53,54]. As a result, greater numbers of motor signals are created and amplified, increasing function and range of motion [55]. Additionally, the prevention of disorganized nerve ends reduces neuroma formation and phantom limb pain [56]. For TMR, at least 7–8 cm of distal radius should be resected to ensure adequate soft tissue coverage and space for MYO prosthetic components [55].

### 4.5. Elbow Disarticulation

#### 4.5.1. Surgical Recommendations

When the radius and ulna cannot be salvaged, elbow disarticulation preserves shoulder rotation and can be performed if the humeral condyle is intact [31]. While typically less preferable than transradial or transhumeral amputations, it is an option when soft tissue coverage is insufficient to retain the radius [57]. It offers a distinct advantage over transhumeral amputation by maintaining internal and external rotation [49]. Additionally, humeral condyle preservation aids in prosthetic suspension and humeral rotation [41,58]. Successful outcomes depend on muscle stabilization and careful contouring of the condyles to prevent pressure intolerance [49]. However, this level is less favored because the current MYO elbows add 5–6 cm to the upper arm, making prosthetic fitting difficult [49,59]. As a result, elbow disarticulation remains controversial, as amputees are often limited to cable controls and weaker external hinges [60].

#### 4.5.2. Prosthetist Recommendations

Achieving limb length symmetry is challenging at this level due to intact condyles and the longer total limb compared to the contralateral side. Traditional elbow units use a turntable mechanism for humeral rotation, but these amputations require outside locking hinges to lock and unlock the elbow joints. This limits the rotation of the elbow and increases the width [47]. A hybrid prosthesis is preferred if the patient desires more advanced functionality than is possible with the conventional BP unit. This uses outside locking hinges paired with a MYO or multi-articulating hand using a quick-disconnect wrist unit. For those patients who are seeking improved limb length symmetry or wish to use a powered elbow, a humeral midshaft bone shortening osteotomy can be performed. This involves removing about 5 to 6 cm of bone from the midportion of the humerus. Although technically demanding, it preserves the humeral condyles, aiding in prosthetic suspension, and allows for some retained rotational movement [59].

### 4.6. Transhumeral Amputation

#### 4.6.1. Surgical Recommendations

For transhumeral amputations, residual limb length impacts prosthetic use and fit. Retaining a minimum of 5–7 cm or 25–30% of the humeral length is recommended for optimal fitting [48]. This helps with weight distribution and maintains shoulder/upper arm contour for clothing [25]. Preserving the deltoid, pectoralis major, and latissimus dorsi insertions maximizes range of motion [49]. Sharp humeral edges should be removed.

#### 4.6.2. Prosthetist Recommendations

**Figure 6:**
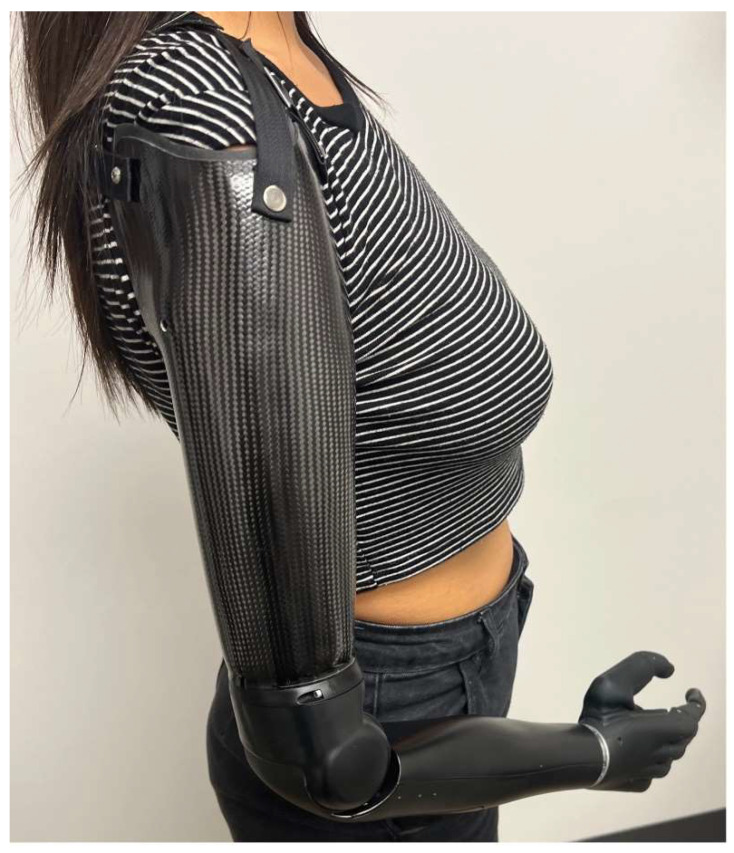
Transhumeral myoelectric prosthesis.

A mid-length amputation allows for most prosthetic options, which usually include either a BP or MYO elbow (Figure 6). The optimal amputation site is determined by [47]:

*Y* = *X* − 14 cm (2)

where Y is the optimal residual limb length (including soft tissue) from the acromion, X is the distance from acromion to distal olecranon (unaffected limb, elbow at 90°), and 14 cm is the average prosthetic elbow unit length.

Prosthetists recommend a hybrid system with a BP elbow and electric lock, which improves efficiency, allowing faster elbow positioning and simultaneous MYO hand control. Harnessing, cabling, and suspension of the prosthetic socket must be maintained, and can enhance prosthetic suspension and rotational control by reshaping the distal humerus. This entails removing a wedge-shaped piece of bone to create an angled portion at the distal humerus. By adjusting the angle of the residual humerus and creating a more anatomically favorable shape, the prosthesis can have better alignment and rotational control, while preserving length. As stated above, a midshaft bone shortening osteotomy of an elbow disarticulation may be performed to aid in rotational control [59].

### 4.7. Shoulder Disarticulation

#### 4.7.1. Surgical Recommendations

Shoulder disarticulation is performed for tumors or severe injury when the humerus cannot be salvaged [2,61]. To improve cosmesis, the scapula is retained, and in female patients, deltoid preservation helps maintain breast contour [2]. Deltoid preservation is also essential for optimal MYO prosthetic control [2]. The rotator cuff is sutured over the glenoid fossa, while the deltoid is secured to the inferior glenoid and lateral scapular border to fill the subacromial space [2]. After securing all muscles in the glenoid cavity, the flaps are smoothed to refine the shoulder contour [2].

To maximize potential for TMR, the musculocutaneous, median, ulnar, and radial nerves and brachial plexus are dissected [41]. Nerve pairings include the musculocutaneous nerve to the clavicular head of the pectoralis major, the median nerve to the motor nerve of the sternal head of the pectoralis major, the radial nerve to the thoracodorsal or long thoracic nerve, and the ulnar to the sternal head of the pectoralis major or minor [41,62].

#### 4.7.2. Prosthetist Recommendations

**Figure 7:**
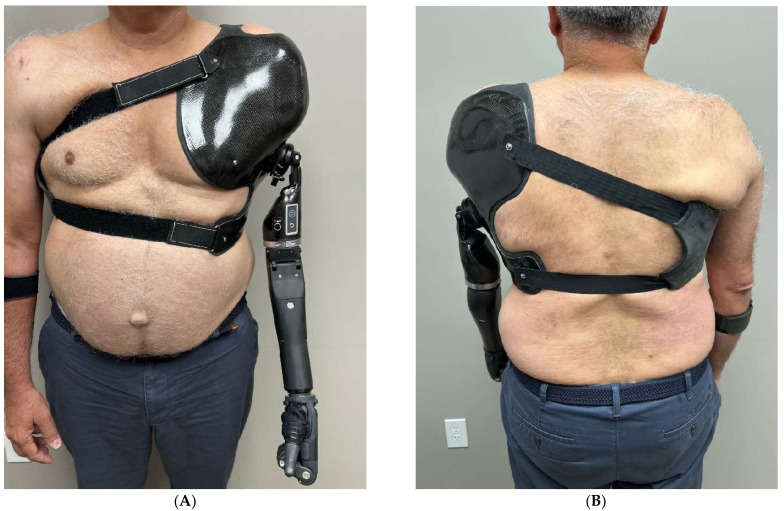
(**A**) Anterior and (**B**) posterior views of X-Frame style socket with cross-body strap system for shoulder disarticulation prosthesis.

One of the most challenging prostheses to fit and wear is a shoulder disarticulation prosthesis. Weight, limited ROM, and battery needs lead many patients to forgo long-term use. An X-Frame socket with a cross-body strap system is recommended for suspension (Figure 7). MYO devices are commonly used for this amputation length, as you lose half of the excursion necessary to control the elbow and terminal devices. Given that BP prosthetics are of minimal use at this level, TMR should highly be considered to optimize MYO components [63].

To guide future practice, recommendations from both surgeons and prosthetists and summaries of the optimal amputation levels for functional prosthetic use are described in Table 3.

## 5. Conclusions

This paper compares the optimal sites of amputation for upper extremities taught to surgeons with those recommended by prosthetists to enhance function and quality of life through effective prosthetic use (Table 3). Early coordination between surgeons, prosthetists, therapists, and social workers is crucial in the preparation for an upper extremity prosthesis after amputation. Prosthetists take a personalized approach to amputation site selection, considering functional challenges from limb length asymmetry and individual patient preferences. As prosthetic technology advances, their focus has shifted toward achieving symmetrical limb lengths, factoring in measurements for wrist flexion, socket material, and terminal devices. They also prioritize patient-specific needs, recognizing that task importance varies among individuals. A major challenge in upper extremity prosthesis engineering arises when striving for symmetrical limb lengths. While surgeons emphasize maximal limb preservation, prosthetists recommend a tailored approach that accounts for contralateral limb length and prosthetic dimensions. This requires surgeons to consider not only the terminal device but also socket components and other prosthetic elements. Despite recommendations, selecting the optimal amputation site has limitations. Surgeons’ choices are often constrained by the extent of the residual limb after trauma, affecting the generalizability of findings. Prosthesis recommendations may also be biased by prosthetists’ preferred devices and the populations they serve. To reduce this bias, future research should include prosthetists from diverse regions and backgrounds. Collaboration among surgeons, prosthetists, and occupational therapists—starting preoperatively—is crucial for better outcomes. Additionally, input from various prosthetic companies is needed for a more comprehensive understanding. Future studies should address these challenges to advance the field.

## Figures and Tables

**Table 1:** Differences in the types of prosthetic devices.

Type	Advantages	Drawbacks	Examples
Passive Functional Prosthetics (PFPs)	High cosmesis Waterproof Lightweight	Minimal function	Vincent Passive Thumb, Titan Thumb, M-Thumb, Regal Prosthesis Ltd., Livingskin, Point Partial, Point Digit, Grip Lock, Point Digit, Grip Lock, Titan Full, Vincent Passive
Body-Powered (BP) Prosthetics	Durable Waterproof	Low cosmesis Heavy Lower fine motor control	Thumb Driver, PIP Driver, MCP Driver, Partial M-Finger, Robin-Aids, M-Fingers
Myoelectric (MYO) Prosthetics	Fine motor control Moderate cosmesis Lightweight	Fragile Expensive	Ossur iDigits, Vincent Partial Hand

**Table 2:** Common terminal device measurements.

Type of Terminal Device	Prosthetic Brand	Available Sizing
Myohands	COVVI	Small: 6.75″, Medium: 7.75″, Large: 7.75″
	Bebionic	Small: 6.50″, Medium: 7.50″, Large: 7.875
	Taska	7.75″ or 8.25″
	Taska CX	7.25″
Body-Powered Hook	Hosmer 5x	Adult: 4.9″
	Hosmer 99x	Adult Small: 3.9″
Myo Elbows	Utah Power U3	2.5″ to elbow center, 4″ to ulnar surface
	Steeper Espire	1.89″
	Motion Arm	1.5″

**Table 3:** Summary of surgical recommendations based on amputation site.

Site of Amputation	Prosthetics Recommended	Minimum Residual Length	Maximum Residual Length	Other Considerations
Thumb	PFP or BP prosthesis	Preserve as much length as possible		
Phalanges Distal to DIP	PFP	Preserve as much length as possible		
Phalanges Proximal to DIP	PFP or BP prosthesis	Consider more proximal mid-phalanx amputation over disarticulation for digit symmetry	Ensure adequate soft tissue coverage to prevent painful stumps	Ray amputation can be considered in cases with central digit involvement
Metacarpals	PFP, BP, or MYO	Must retain enough length for functional grip	Consider thin flap coverage	Starfish procedure may be performed if MYO device desired
Wrist	PFP, BP, or MYO prosthesis with low-profile wrist unit	Not ideal for prosthetics due to limb length discrepancy		Preserve ulnar styloid and extensor tendon
Radius	BP or MYO prosthesis	Retain at least 4 cm of the ulna from the olecranon	Must allow room for terminal device + socket (~22.5 cm from lateral epicondyle)	If TMR is desired, at least 7–8 cm of distal radius should be resected
Elbow	BP or hybrid design	Not ideal for prosthetics due to limb length discrepancy		Humeral midshaft osteotomy can improve symmetry and control while preserving humeral condyles
Humerus	Hybrid design with BP elbow and myoelectric hand	Retain at least 5–7 cm (or 25–30%) of humeral length from the acromion	Must allow room for terminal device + socket (~14 cm from acromion)	Humeral angle or midshaft osteotomy can improve suspension and rotational control
Shoulder	MYO prosthesis with X-Frame socket	Preserve scapula and deltoid for shoulder contour and prosthetic control	Ensure adequate soft tissue coverage for prosthetic suspension	TMR should be highly considered

## Data Availability

No new data were created or analyzed in this study.
